# Limb Occlusion Pressure Versus Standard Pneumatic Tourniquet Pressure in Open Carpal Tunnel Surgery – A Randomized Trial

**DOI:** 10.7759/cureus.20110

**Published:** 2021-12-02

**Authors:** Hannah Morehouse, Haley M Goble, Bradley S Lambert, Jaclyn Cole, Brendan M Holderread, Jessica T Le, Todd Siff, Patrick C McCulloch, Shari R Liberman

**Affiliations:** 1 Orthopedics and Sports Medicine, Houston Methodist Hospital, Houston, USA

**Keywords:** standard pneumatic tourniquet, limb occlusion pressure, post operative pain management, carpal tunnel, opioid use, hand surgery

## Abstract

Introduction: Pneumatic tourniquets are used extensively in orthopedic hand/wrist surgery. Complications, while rare, are associated with elevated pressure and duration of tourniquet use. Limb occlusion pressure (LOP) is the minimum tourniquet pressure at which arterial blood flow is restricted. Therefore, we performed a cross-sectional double-blinded randomized control trial to assess if there is a difference in post-operative pain at the surgical and tourniquet site between LOP and standard tourniquet pressure and if there is a difference in post-operative opioid usage.

Methods: A total of 44 patients (Age 60±13, 30 female, 14 male) were randomized into two groups (LOP, 191±14 mmHg | STP, 250 mmHg) of 22 patients controlling for gender (15 female, seven male). The primary outcome was a visual analog scale (VAS) for pain at the tourniquet and surgical sites, recorded for the first two weeks post-operative. Daily pain medication usage was recorded and quantified using oral morphine milligram equivalents (MME). A group-by-time generalized mixed-model ANOVA was used to detect within-group and between group (LOP vs STP) differences in VAS at the surgical and tourniquet sites as well as medication use.

Results: LOP significantly decreased post-operative pain medication usage across the first week (-50%; p<0.05). Both groups had similar VAS pain at the surgery site, but the LOP group had 80% reduced pain at the tourniquet site when averaged across the first post-operative week (p<0.05).

Conclusions: The use of LOP compared to STP elicits reduced post-operative pain at the tourniquet site and reduces post-operative pain medication use in the first post-operative week.

## Introduction

Modern advances in tourniquet use include ease of use, numerous safety features, and the limb occlusion pressure (LOP) function [1-3]. Tourniquet pressures range from 200-350 mm Hg depending on location [4]. Elevated tourniquet pressures have been associated with increased post-operative pain and increased risk of complications, which can include skin irritation and abrasions, nerve or muscle injury, ischemia, and bruising [4,5]. In rare instances these lead to severe morbidity and mortality secondary to tourniquet use [6-8]. These deleterious side effects indicate a need for individualized tourniquet pressures.

LOP is defined as the tourniquet pressure at which arterial blood flow is occluded [1]. Modern tourniquet systems utilize a distal photoplethysmography probe that functions using a light transducer in coordination with a gradually inflating tourniquet cuff to determine at which inflation pressure distal blood flow is occluded [9,10]. Establishing LOP requires attaching the photoplethysmography probe to the distalmost aspect of the extremity requiring tourniquet use and running the preset system for LOP measurement, which is a component of modern pneumatic tourniquets. Once this data point is established, the tourniquet system will then be set to inflate at this pressure. Previous studies have demonstrated that LOP provides an effective hemostatic surgical field without increased bleeding compromising visualization similar to standard tourniquet pressures (STP) in total knee arthroplasty and anterior cruciate ligament (ACL) reconstructions [3,11,12]. However, because pressures are normalized to each individual with LOP, the risk of tourniquet-associated site pain and bruising as a result of over-pressurized cuffs may be reduced [3,11]. For example, Reilly et al. [12] were able to decrease mean cuff pressure from 300 mm Hg to 151 mm Hg using LOP without causing a detrimental effect on the bloodless quality of the operative field in ACL surgery. 

Most existing tourniquet literature is based on lower extremity data; however, upper extremity surgeons rely on tourniquets to maintain visualization throughout surgical procedures. There have been recent attempts at minimizing tourniquet pressures in upper extremity surgery through multiple methods, including LOP, systolic blood pressure (SBP) measurements, and doppler ultrasound [2,9,13]. These methods are employed to decrease the risk of complications, including abrasions, bruising, and nerve damage, which can be quite significant. Although complication risks are low, any complication after an elective upper extremity procedure, such as carpal tunnel release, can delay a patient’s return to optimal functional status.

Postoperative pain control is fraught with many confounding factors, including psychosocial factors, patient perception, operative technique, and can also have an effect on surgical outcome and patient satisfaction [14-16]. Relatedly, it is difficult to predict the dosage for different patients post-operatively, which can lead to over-prescribing medications to maintain overall patient satisfaction. Due to the rising opioid epidemic in the United States (US), orthopedic surgeons and surgical procedure selection play an important role in minimizing post-operative opioid prescriptions. 

The primary purpose of this study was to determine if LOP causes less post-operative pain than STP at both the tourniquet and surgical sites following open carpal tunnel release. In conjunction with this aim, our secondary aim was to determine if potential reductions in post-operative pain translated into reduced opioid medication use for a period of two weeks following surgery. In light of previous literature, we hypothesized that LOP would decrease post-operative pain at the tourniquet site and overall narcotic usage. Additionally, we hypothesized that patient outcomes would be improved in the LOP group by decreasing complications and adverse side effects while having no effect on intraoperative blood loss or impairment of surgeon visibility.

## Materials and methods

Recruitment

**Figure 1:**
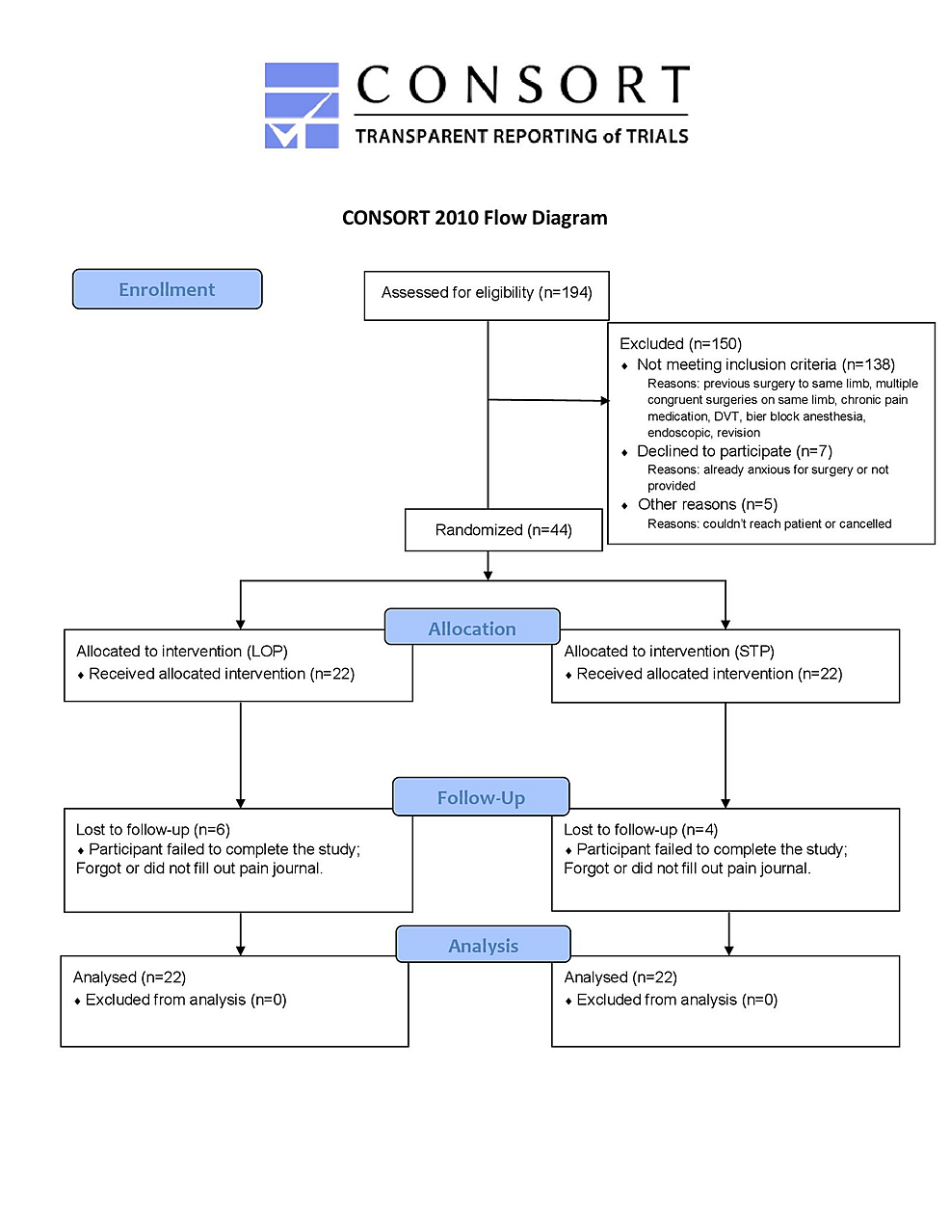
CONSORT 2010 Flow Diagram Adapted from: CONSORT 2010 Statement

Inclusion criteria consisted of all patients >18 years old undergoing open carpal tunnel release by one of two fellowship-trained orthopedic hand surgeons for mild, moderate, or severe carpal tunnel syndrome. The patient's diagnosis was confirmed via clinical assessment and/or electromyographic testing that failed non-operative treatment [17]. Patients were excluded for the following conditions: currently taking prescription pain medications for chronic conditions (>6 weeks), patients who cannot use a tourniquet (i.e. fistulas, peripheral vascular disease (PVD)), prior trauma or surgery to the intended limb, and hypo- or hypertension preventing accurate distal photoplethysmography probe reading. The study followed the Consolidated Standards of Reporting Trials (CONSORT) 2010 checklist for reporting of randomized controlled trials (Figure 1).

All patients were randomized using a digital randomization function (Microsoft Excel™, Microsoft Corporation, Redmond, Washington) to either the LOP or STP group. A total of 44 patients (Age 60±13yr, 30 female, 14 male) completed all aspects of the study between January 1, 2019 - January 1, 2020 (STP: n=22, m=7, f=15 | LOP: n=22, m=7, f=15).

Surgical procedure - open carpal tunnel release

All procedures were performed by two fellowship-trained orthopedic hand surgeons with standardized operative techniques. Surgeons and patients were blinded to the tourniquet technique using a cover over the pneumatic tourniquet controls and all necessary measurements were obtained prior to the surgeon entering the operating room. A tourniquet was placed on the operative extremity. The operative extremity was prepped and draped in a sterile fashion typical of standard surgical procedures. An Esmarch was used to exsanguinate the limb and the tourniquet was inflated to either STP (250 mm Hg) or LOP, which was premeasured, unique to each patient, and determined prior to surgery. Systolic blood pressure (SBP) and tourniquet pressure were recorded. Anesthesia was performed based on patient characteristics, preference, and anesthesiologist comfort. Patients had either general anesthesia or local/monitored anesthesia care (MAC) anesthesia.

Both surgeons performed standard open carpal tunnel releases through a small volar incision. At the end of the case, all patients had 10cc of 0.5% bupivacaine injected at the surgical site. Wound closure was managed per surgeon preference with either interrupted 4-0 nylon or 4-0 subcuticular Monocryl. During surgery, intraoperative blood loss or impairment of surgeon visibility was rated on a scale of 0 (poor visibility) - 4 (excellent visibility) (Supplement) [18].

Pre and post-operative measurements

Prior to participating, all patients were required to complete the disabilities of the arm, shoulder, and hand (DASH) questionnaire regarding functional disabilities of the hand [19]. Throughout the course of the study, patients kept a daily pain diary using a VAS for pain at the tourniquet site (three times per day: morning, mid-day, evening) for two weeks. These pain diaries were then submitted at the two-week post-operative appointments. Daily narcotic and over-the-counter (OTC) pain medication usage was also recorded for the initial two weeks after surgery. All narcotic medication usage was standardized and quantified via the morphine milligram equivalents (MME) conversion, which allowed for comparison between medications [20]. Patients answered a series of binary questions including bruising, blistering, tourniquet pain. The same DASH questionnaires were completed at the two-week and eight-week post-operative clinic visits. Surgeons were also queried regarding intraoperative blood loss and any visual field difficulties during the case. 

Statistical analysis

Power and sample size: Previous literature [21] and preliminary analysis pilot data (N=20, LOP (10), STP (10)) were used to determine sample size. For a within-group change of 1.4 VAS (Minimum clinically important difference) [21] as our primary outcome variable and 10% difference in opioid usage (secondary outcome variable) for a power of 0.8 at a type I error threshold of α=0.05, it was determined that 22 participants would be required for each treatment group (LOP and STP).

A mixed-model analysis of covariance (ANCOVA) repeated on time, co-varied on baseline values was used to compare patient recorded VAS pain scores and medication usage (MME units, acetaminophen, ibuprofen, naproxen) between groups (LOP and STP) for 14-days post-surgery. For all significant interactions indicated by type III tests of fixed effects, a Tukey’s post hoc adjustment was used for within and between group pairwise comparisons. Average pain medication usage and pain scores were compared using a two-tailed independent samples t-test for the first 48h post-op and across seven days post-op. A Mann-Whitney test for non-parametric data was used to compare the intra-operative visibility scores. Lastly, a Fisher’s exact test was used to compare the proportions of each subject group that reported taking any medication or having a VAS recorded pain for each day of the post-operative period. Type I error for all comparisons was set at α=0.05. For significant pairwise comparisons between groups, effect size was calculated using a Cohen’s D statistic. Effect sizes (ES) are interpreted as follows: 0-1 (N, negligible); 1-3 (S, small); 3-5 (M, moderate); 5-7 (L, large); >7 (VL, very large).

## Results

Results for DASH scoring, intra-surgery measures, and post-operative blistering/bruising are as follows: no differences detected between groups other than tourniquet pressure used (p<0.001), no differences were observed between groups for OTC medication use for any of the post-operative comparisons, and level of carpal tunnel severity did not differ between groups (STP: Mild (n=9), Moderate (n=12), Severe (n=9) | LOP: Mild (n=10), Moderate (n=9), Severe (n=10)) (Table 1).

**Table 1:** Functional outcomes and intra-operative measurements STP: standard tourniquet pressure; LOP: limb occlusion pressure; SBP: systolic blood pressure; DBP: diastolic blood pressure; ns: not significant Values are presented as means ± 95%CI.  For DASH scoring, like letters are not significantly different within group from timepoint to timepoint.  Type I Error set at (α=0.05)

Disabilities of the Arm, Shoulder, and Hand (DASH) Score			
	STP	LOP	Sig. (Between Group)
Pre-Op	61 ± 10	61 ± 10	ns
2 Weeks Post-Op	49 ± 6	52 ± 8	ns
8 Weeks Post-Op	43 ± 8	36 ± 3	ns, P=0.07
Intra-Surgery Data			
	STP	LOP	
Tourniquet time (min)	7 ± 2	8 ± 2	ns
Tourniquet Pressure (mmHg)	250	191 ± 14	P<0.001
Blood Pressure (mmHg)			
SBP	133 ± 9	125 ± 8	ns
DBP	69 ± 3	68 ± 4	ns
Quality of Bloodless Scale (0-4)	3.7 ± 0.2	3.7 ± 0.2	ns
Bruising at the tourniquet site?	0%	0%	ns
Blistering at the tourniquet site?	13%	13%	ns

Post-operative pain

**Figure 2:**
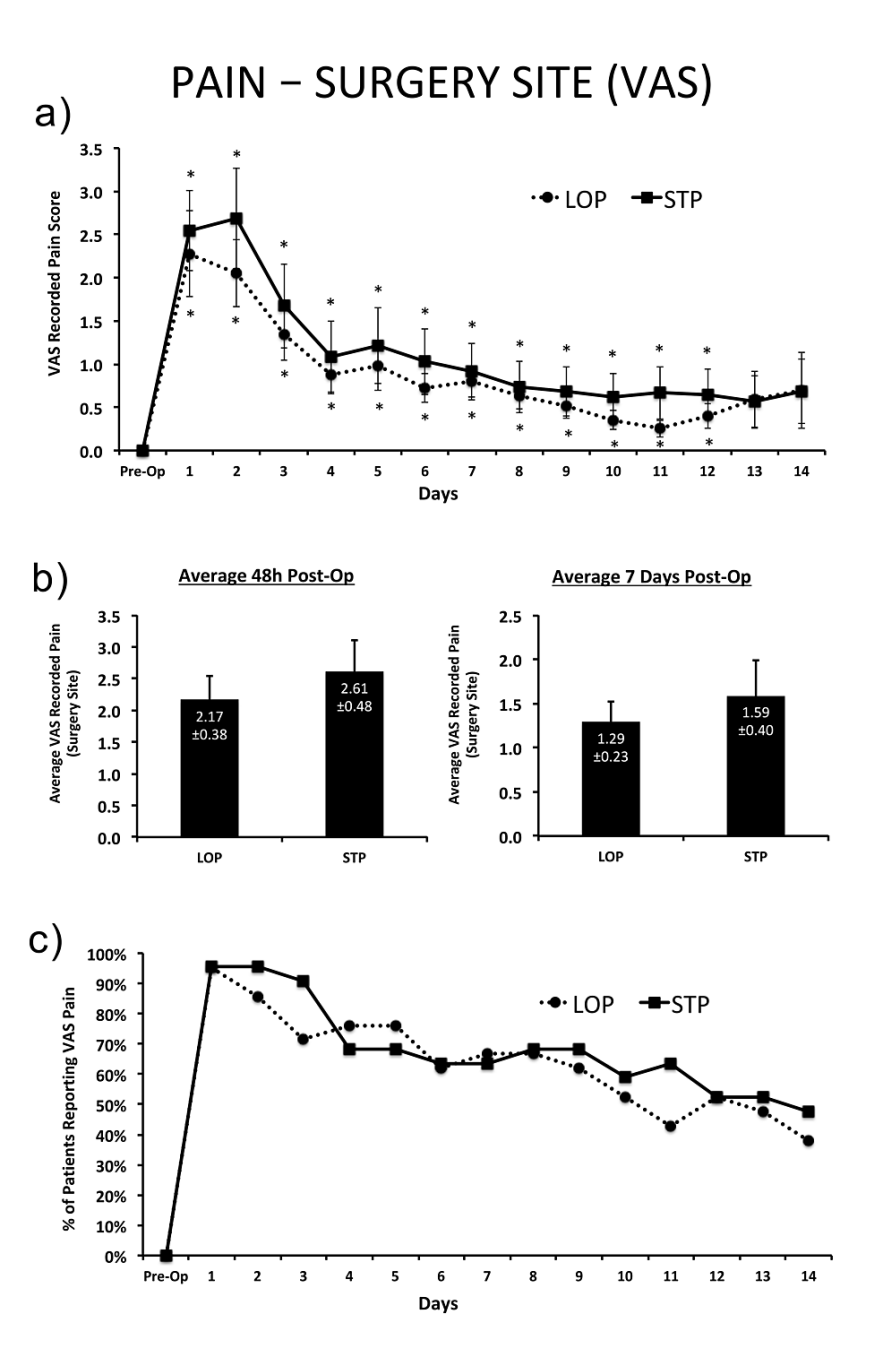
Surgical site pain using VAS for (a) each day post-surgery out to 14 days post-op; (b) averaged across the first 48h and 7 days post-op; and (c) the percentage of patients within each group reporting any pain within the first 14 days post-op. VAS: visual analog scale * = Significant difference from pre-op baseline within group; †=significant difference between groups at the same measurement time-point. Effect sizes (ES) are interpreted as follows:  0-1 (N, negligible); 1-3 (S, small); 3-5 (M, moderate); 5-7 (L, large); >7 (VL, very large). Type I error set at α=0.05. Data are presented as means±SEM.

There was no significant difference between LOP and STP groups in regard to pain at the surgery site with both groups having similar post-operative pain responses (Figure 2).

**Figure 3:**
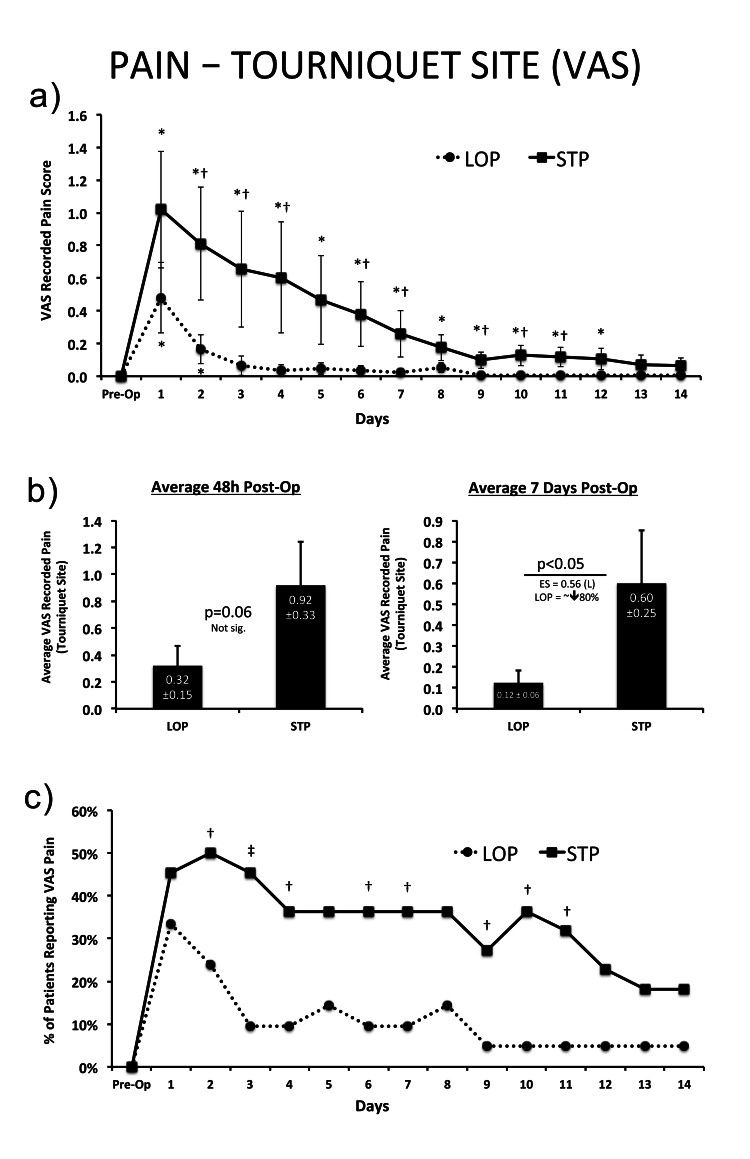
Tourniquet site pain using VAS for (a) each day post-surgery out to 14 days post-op; (b) averaged across the first 48h and 7 days post-op; and (c) the percentage of patients within each group reporting any pain within the first 14 days post-op. VAS: visual analogue scale * = Significant difference from pre-op baseline within group; †=significant difference between groups at the same measurement time-point. Effect sizes (ES) are interpreted as follows:  0-1 (N, negligible); 1-3 (S, small); 3-5 (M, moderate); 5-7 (L, large); >7 (VL, very large). Type I error set at α=0.05. Data are presented as means±SEM.

However, tourniquet site pain was reduced with the use of LOP compared to STP. The LOP group reported decreased post-operative pain relative to the STP group at several time points (Days 2-4,6,7,9-11, p<0.05). This amounted to approximately an 80% reduction when pain scores were averaged across the first seven days post-surgery (p<0.05). Of note, post-operative VAS scores returned to being not significantly different from “0” by three days post-op in the LOP group compared to day 13 in the STP group. The proportion of patients logging a pain score greater than zero was observed to be significantly higher in the STP group at days 2-4,6,7, and 9-11 post-surgery (p<0.05) (Figure 3).

Opioid medication use

**Figure 4:**
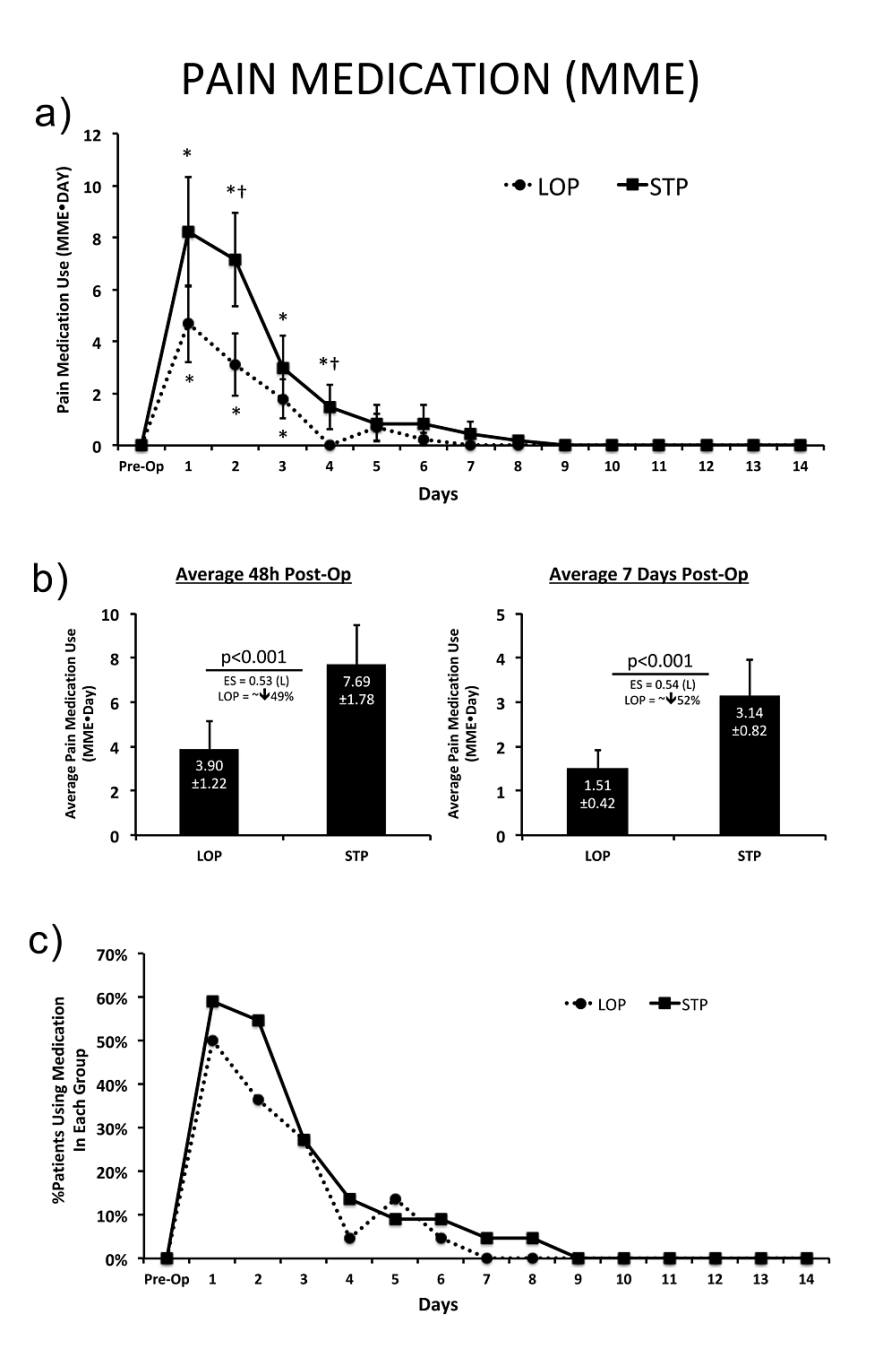
The 14-day post-operative pain medication use, as measured by MME. (A) opioid pain medication use for each day post-surgery to 14 days post-op; (B) averaged across the first 48h and seven days post-op; and (C) the percentage of patients within each group reporting to take pain medication within the first 14 days post-op. Opioid pain medication use calculated as morphine milligram equivalence (MME). * = Significant difference from pre-op baseline within group; †=significant difference between groups at the same measurement time-point. Effect sizes (ES) are interpreted as follows:  0-1 (N, negligible); 1-3 (S, small); 3-5 (M, moderate); 5-7 (L, large); >7 (VL, very large). Type I error set at α=0.05. Data are presented as means±SEM.

The LOP group consumed significantly less pain medication across the first week post-surgery (p<0.05). This was observed at individual post-operative days (POD) 2 and 4 (p<0.05). This was also observed when looking at average consumption across the first 48 hours (p<0.001) post-operatively as well as the average for the first seven days post-operatively (p<0.001, ~50% reduction). Although no difference was detected for the proportion of patients taking pain medications each day, all patients in the LOP group were found to be finished taking medication by POD 7 whereas all patients in the STP group were observed to be finished by POD 9 (Figure 4). 

## Discussion

The primary goal of this study was to determine if the use of LOP results in less post-operative pain than STP during open carpal tunnel release. Second, we sought to determine if reductions in post-operative pain would be associated with decreased post-operative opioid usage. When used in carpal tunnel surgery, the use of LOP significantly reduced the pain at the tourniquet site in the LOP group with a concomitant decrease in medication taken by approximately 50% during the first week following surgery. Patient pain outcomes at the surgery site showed no difference between LOP and STP. There was also no difference in the bloodless quality of the surgical field suggesting that the use of LOP did not visually impact the procedure from the surgeon’s point of view intraoperatively. Cumulatively, these results provide considerable support for the use of LOP in carpal tunnel release and potentially other hand surgeries for improving patient reported outcomes in the early post-operative period. Importantly, while the use and frequency of opioid medication abuse may not be as prevalent for smaller-scale procedures such as carpal tunnel release, the present findings provide further support for investigating the use of LOP in more invasive surgeries such as ACL reconstruction or total-joint replacement.

Although adequate data are still lacking in the literature, LOP has been analyzed in a few other instances within orthopedic surgery. McEwen et al. [1,11] showed that the use of LOP can decrease thigh tourniquet pressure by 19-42%. Additionally, Reilly et al. [12] showed no difference in the bloodless quality of the operative field in anterior cruciate ligament reconstruction. This has also been extended to total knee arthroplasty (TKA), which has also shown a decrease in typical cuff pressure by 33-42% when using limb occlusion pressure and wide contoured cuffs [3]. Olivecrona et al. [22] found decreased wound complications following TKA when using LOP and tourniquet pressures <225 mm Hg. However, to our knowledge, this study is among the first to examine the use of LOP versus STP with regards to examining post-operative tourniquet site pain and opioid consumption.

Tourniquet site pain and risk

Risks associated with tourniquet use can include neurologic injury, abrasions, bruising, and pain. These complications can be worse than at the surgical site [2,10,23]. Muscle and nerve injuries are the most worrisome complications due to their severity [23,24]. Complication rates increase with prolonged tourniquet use, elevated tourniquet pressure, and improper skin padding [25]. While the present study involved relatively short tourniquet times (~7-8min) the present findings are encouraging for potentially preventing such injuries in longer duration procedures.

Reduction in pain medication usage

Surgeons are often blamed as being the “gatekeepers” for opioid addiction due to excessive prescribing. However, there are minimal guidelines to assist with outpatient pain management, potentially leading to iatrogenic opioid abuse [16]. Pain is difficult for prescribers to predict following surgery since it has multiple underlying psychological and physiologic factors [14,15]. However, orthopedic surgeons often prescribe many more narcotics than needed. Chapman et al. [26] performed a prospective study showing an over-prescription of narcotic medications at an average of 5:1. In the present study, the greatest reductions in pain and medication use occurred within the first four to seven days post-surgery when pain ratings were the highest for both groups (particularly in the initial 48 hours post-surgery). Therefore, ameliorating pain during this time period is of significant interest with regards to physical and psychological habit-forming [14]. In the present study, the use of LOP significantly decreased post-operative narcotic use. This finding suggests that LOP may potentially produce similar beneficial outcomes in other surgeries requiring tourniquets, thereby decreasing the number of opioid medications prescribed by orthopedic surgeons. Most patients take an average of 4.9 pills for the first 2.3 days post-operatively [27]. Therefore, although this procedure does not necessitate large amounts of opioid prescriptions, it is worthwhile to minimize narcotics wherever possible.

Clinical relevance

Minimally clinically important difference (MCID) for VAS can range from 1.2-5.4 depending on surgery type [28,29]. When analyzed at individual time points, while this threshold may not have been reached between groups, there were prolonged improvements in VAS for LOP vs STP over time. The duration of pain in the STP group vs the LOP group was also prolonged. The primary evidence to suggest these reductions in pain were clinically meaningful can be seen in the ~50% decrease in post-operative narcotic use over the first week post-operative in the LOP group compared to the STP group. While post-operative pain following open carpal tunnel release is generally low relative to more invasive surgery types, the present data indicate that tourniquet pain does factor into decision-making with regards to whether or not patients elect to take medications. This was demonstrated as patients in both groups had similar surgical site pain but the LOP group had reduced tourniquet site pain. The fact that these reductions mirrored reduction in medication use suggests that the use of LOP did have a significant overall impact on patient outcomes. Whether or not tourniquet pressure factors into site pain to a degree that influences medication use in other surgery types represent topics of future investigation.

Limitations

This study is not without limitations. This investigation is a single surgery type with relatively short tourniquet times. However, patients were able to show a statistically significant difference in tourniquet site pain between LOP and STP despite such short tourniquet times, which is encouraging for future studies involving longer tourniquet times and higher post-operative pain levels. Patient pain tolerance variability is a limitation in any study analyzing pain medication usage post-operatively. Self-reporting also has inherent limitations affecting data interpretation [30]. Additionally, patients may misuse pain medications, as patients in some studies have reported taking medication due to psychological factors instead of physical pain [14,15]. The randomized nature of the present study may help to overcome this factor.

## Conclusions

LOP can be applied to upper extremity orthopedic surgery, particularly carpal tunnel release, in a clinically impactful way that may decrease post-operative tourniquet site pain and opioid use. Most modern pneumatic tourniquet systems in operative centers have a LOP function and can be used with relative ease and minimal additional preparation time by operating room staff prior to incision. There is very little to no added risk or additional cost in the operating room when utilizing the LOP technology and is, therefore, an excellent candidate for becoming standard of care for reductions in post-operative pain and medication use. Further study is required to determine if LOP provides benefit in other surgical procedures where post-operative pain and narcotic use are common.
